# Statement of Retraction

**DOI:** 10.1080/10717544.2022.2157537

**Published:** 2023-01-18

We, the Editors and Publisher of the journal *Drug Delivery* have retracted the following article:

Yanyan Qi, Xiangyan Yao, Xianhui Du & Songtao An (2021) Local anesthetic lidocaine-encapsulated polymyxin–chitosan nanoparticles delivery for wound healing: in vitro and in vivo tissue regeneration, Drug Delivery, 28:1, 285-292, DOI: 10.1080/10717544.2020.1870021

Since publication, concerns have been raised about the similarity of the abstract, experimental section, results and discussion, and conclusion to another article published in the journal:

Lianlian Cao, Guojing Shao, Fengmei Ren, Minghua Yang, Yan Nie, Qian Peng & Peng Zhang (2021) Cerium oxide nanoparticle-loaded polyvinyl alcohol nanogels delivery for wound healing care systems on surgery, Drug Delivery, 28:1, 390-399, DOI: 10.1080/10717544.2020.1858998

During the investigation, additional concerns regarding animal welfare and ethical approval were observed. The authors were contacted for an explanation but did not reply. As a result, we are retracting the article. The corresponding author listed in this publication has been informed.

We have been informed in our decision-making by our policy on publishing ethics and integrity and the COPE guidelines on retractions.

The retraction article will remain online to maintain the scholarly record, but it will be digitally watermarked on each page as ‘Retracted’.

